# Olive Oil Traceability Studies Using Inorganic and Isotopic Signatures: A Review

**DOI:** 10.3390/molecules27062014

**Published:** 2022-03-21

**Authors:** Emna G. Nasr, Ekaterina N. Epova, Mathieu Sebilo, Dominic Larivière, Mohamed Hammami, Radhia Souissi, Houyem Abderrazak, Olivier F. X. Donard

**Affiliations:** 1 Institut des Sciences Analytiques et de Physicochimie pour l’Environnement et les Matériaux, Université de Pau et des Pays de l’Adour, 64000 Pau, France; mathieu.sebilo@sorbonne-universite.fr (M.S.); olivier.donard@univ-pau.fr (O.F.X.D.); 2 Laboratoire des Matériaux Utiles, Institut National de Recherche et d’Analyse Physicochimique Technopole de Sidi Thabet, Ariana 2020, Tunisia; mohamed.hammami@inrap.rnrt.tn (M.H.); souissiradhia@yahoo.fr (R.S.); houyem.snani@yahoo.fr (H.A.); 3 Faculty of Sciences, Farhat Hached Universitary Campus, University of Tunis El Manar, Tunis 1068, Tunisia; 4 Advanced Isotopic Analysis Hélioparc, 64000 Pau, France; ekaterina.epova@ai-analysis.com; 5 Sorbonne Université, Institut d’Ecologie et des Sciences de l’Environnement de Paris (IEES Paris), CNRS, 75005 Paris, France; 6 Département de Chimie, Université Laval, Québec, QC G1V 0A6, Canada; dominic.lariviere@chm.ulaval.ca

**Keywords:** olive oil, geographical authentication, trace elements, stable isotopes of light elements, ^87^Sr/^86^Sr, sample preparation, detection techniques, statistical data treatment

## Abstract

The olive oil industry is subject to significant fraudulent practices that can lead to serious economic implications and even affect consumer health. Therefore, many analytical strategies have been developed for olive oil’s geographic authentication, including multi-elemental and isotopic analyses. In the first part of this review, the range of multi-elemental concentrations recorded in olive oil from the main olive oil-producing countries is discussed. The compiled data from the literature indicates that the concentrations of elements are in comparable ranges overall. They can be classified into three categories, with (1) Rb and Pb well below 1 µg kg^−1^; (2) elements such as As, B, Mn, Ni, and Sr ranging on average between 10 and 100 µg kg^−1^; and (3) elements including Cr, Fe, and Ca ranging between 100 to 10,000 µg kg^−1^. Various sample preparations, detection techniques, and statistical data treatments were reviewed and discussed. Results obtained through the selected analytical approaches have demonstrated a strong correlation between the multi-elemental composition of the oil and that of the soil in which the plant grew. The review next focused on the limits of olive oil authentication using the multi-elemental composition method. Finally, different methods based on isotopic signatures were compiled and critically assessed. Stable isotopes of light elements have provided acceptable segregation of oils from different origins for years already. More recently, the determination of stable isotopes of strontium has proven to be a reliable tool in determining the geographical origin of food products. The ratio ^87^Sr/^86^Sr is stable over time and directly related to soil geology; it merits further study and is likely to become part of the standard tool kit for olive oil origin determination, along with a combination of different isotopic approaches and multi-elemental composition.

## 1. Introduction

Olive oil is a natural product obtained from olives. It is a key component of the Mediterranean diet. Sometimes called “liquid gold”, olive oil is mainly appreciated for its flavour and high nutritional value, along with its recognised potential in the prevention of certain diseases [1,2]. It is also an interesting ingredient in dermo-cosmetology, as it has beneficial effects on the skin [3,4].

Large amounts of olive oil are annually produced around the Mediterranean basin and consumed worldwide. According to the International Olive Oil Council, around 10.2 million hectares of land are devoted to olive growing around the world, and the world production of olive oil from IOC member countries has tripled in the last 60 years. Production has been estimated to be almost 3 million tonnes for the period 2019/2020, with about 2 million tonnes produced in Europe and about 1 million tonnes in non-European countries. (IOC: International Olive Council/olive oil-estimated 2019/2020 crop year). The increased production of olive oil has been driven by increasing global consumption of olive oil over the past two decades. Global consumption reached 2,970,000 metric tonnes in 2019/2020 (Statista: Olive oil consumption worldwide, available at https://www.statista.com/statistics/940491/olive-oil-consumption-worldwide/, accessed on 12 March 2021). This global increase in demand has caused the price to rise, and the high economic value of olive oil has led to widespread fraudulent practices. It is now considered to be one of the most adulterated foods [5,6].

False declaration of geographical origin is among the most prevalent forms of olive oil fraud and is the most challenging to detect. The implications of food fraud can range from acute health effects on consumers to significant economic losses. The global financial agri-food industry loss due to olive oil fraud has been estimated to be between $30 and $40 billion per year (PWC 2016, available at https://press.pwc.com/News-releases/fighting--40bn-food-fraud-to-protect-food-supply/s/44fd6210-10f7-46c7-8431-e55983286e22, accessed on 18 January 2022). Reputable producers also suffer reputation loss when consumers lose confidence in the market due to counterfeited poor-quality products [7].

Therefore, establishing a traceability system in the food industry, in particular in olive oil production, has become a major issue to guarantee the quality and authenticity of food products [8]. In Europe, priority is given to products whose quality is guaranteed by European Union schemes of geographical indication, known as Protected Designation of Origin (PDO), Protected Geographical Indication, and Traditional Specialties Guaranteed [9]. Whether a food product is permitted to use these quality labels is directly related to the geographical provenance and often refers to specific production processes.

The PDO label on olive oil has indeed become a motivating choice criterion for customers since olive oil quality and flavour are linked to where the olives grow and associated with specific production practices [10,11]. As a result, the geographical authentication of olive oil is becoming an important trend in scientific research, particularly in the field of analytical chemistry [12]. Between 1994 and 2020, the number of studies containing keywords related to the geographical authentication of olive oil has increased drastically; there were less than 30 articles in the 1990s and more than 240 in 2020 alone [13].

The choice of appropriate analytical strategies for olive oil characterization is complex. Olive oil is a particularly challenging matrix due to its high fatty composition and viscosity. Its lipid nature frequently hampers the traditional direct sample introduction strategies common for various analytical instruments. Furthermore, during the olive oil production process, which starts with olive growing and concludes with storing the extracted oil, many factors affect its overall chemical composition. First, the olive variety has a direct influence on the fatty acids, sterols, and total polyphenol composition [14]. Second, during the growth of the olive trees, the agricultural practices (irrigation and use of fertilisers) that are used can impact the final chemical composition of the olive oil [15]. Third, after harvesting the olives and during the oil extraction process, potential contaminants and alterations can be involved in the final composition of the oil since the olives are in direct contact with different surfaces throughout the processing.

**Figure 1:**
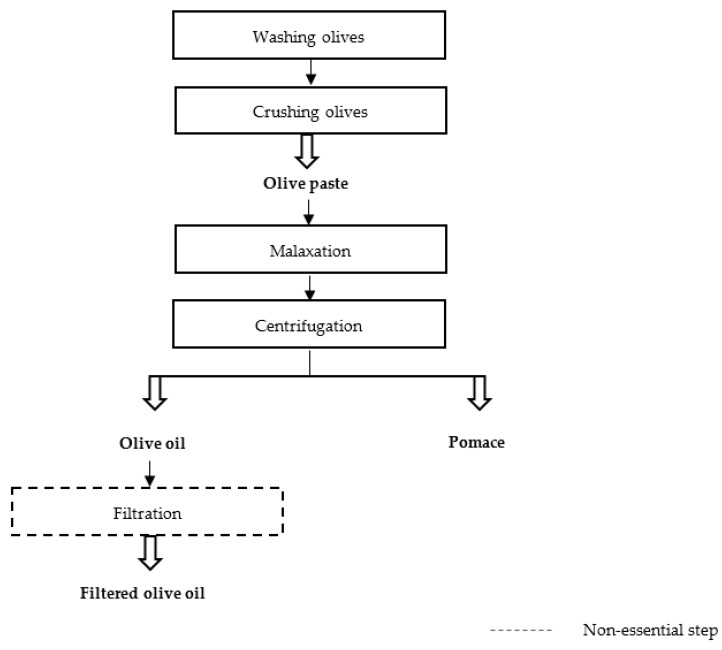
The continuous extraction process of olive oil.

*Effect of olive oil extraction process on its chemical composition*: olive oil is obtained through a multi-stage extraction process composed of several steps. Figure 1 illustrates the various steps of olive oil extraction.

At first, olives are washed and then crushed mechanically to obtain an olive paste. The paste is then subjected to malaxation (churning or milling to aggregate the small oil drops) at ambient temperature. Next, the mixture is centrifuged to separate the olive oil from the pomace. The “olive pomace” is defined as the solid residue recovered during the olive oil extraction process, obtained either by pressing or centrifugation. Pomace includes pieces of skin, pulp, stone, and olive kernel.

Other key parameters that affect the chemical composition of the final product include the oil extraction method [16], the type of oil obtained (virgin, extra-virgin, or lampante) [17], and the filtration of the final product. The shelf life of olive oil also affects the quality of the final product. The current industrial method extends the storage time compared to the traditional pressing method because the industrially extracted oils have a lower percentage of total degraded polyphenols. More recently, it has been shown that the filtration process of olive oil, which mainly removes pomace residues and leftover water, can significantly alter the content of trace elements naturally present in the olive oil [18]. Finally, the storage and transport conditions of the olive oil, if not carefully controlled, affect its oxidative stability [19,20]. Due to all these potential alterations during the olive oil milling process, storage, and transportation, strict analytical control is needed at every single step.

*Organic and inorganic analysis of olive oil*: the analytical methods used for authenticity and traceability assurance of the olive oil should take the various possible changes into consideration in order to obtain controlled and reproducible parameters for overall quality assessment. To respond to such challenging issues, a large array of analytical strategies has been developed. They include organoleptic approaches, biological, chemical, and elemental/isotopic analyses [21]. The usual organic analytical methods are based on the determination of organic compounds such as fatty acids and triacylglycerols [22], phenols [23], sesquiterpene hydrocarbons [24], and sterols [25,26].

The state-of-the-art methods are mostly based on the interpretation of each oil’s elemental profile (as the principal indicator of origin and agricultural practices), stable isotopes of light elements (whose ratio in the final product is primarily linked to the plant growth environment), and the isotopic composition of strontium (as a direct reflection to local soil and geology) [27,28]. Among the various elemental analysis techniques, atomic spectrometry techniques are constantly evolving. These techniques can carry out simultaneous multi-elemental analysis with enhanced resolution, accuracy and sensitivity, which is excellent for the discrimination of food origin [29]. However, olive oil analysis is still challenging as the oil is a fatty matrix with a rich and varied organic content, where chemical elements, other than C, H and O, mainly occur at trace levels ranging from few μg kg^−1^ to few mg kg^−1^ [30]. Therefore, reliable, accurate and precise measurement of trace elements in olive oil is a key issue that requires adequate and efficient sample pre-treatment and preparation methods [18]. A wide range of olive oil preparation techniques has been proposed in the scientific literature. Some preparation methods aim to destroy the organic matter using strong acids (e.g., microwave-assisted digestion, calcination), while others are non-destructive, aiming to extract the elements without significantly altering the matrix (e.g., liquid-liquid extraction, extraction using a chelating agent, sample dilution, emulsification). Trace elements are then quantified by means of several atomic spectrometric techniques (flame atomic absorption spectroscopy: FAAS, graphite furnace atomic absorption spectroscopy: GF-AAS, inductively coupled plasma atomic emission spectroscopy: ICP-OES, inductively coupled plasma mass spectrometry: ICP-MS).

The analysis of stable isotopes, mainly H, C and O isotope ratios, by isotope ratio mass spectrometry (IRMS) and nuclear magnetic resonance (NMR) was also successfully used for geographical discrimination of olive oil, since the isotopic fractionation of these elements is correlated to geographical and climatic parameters [31,32]. More recently, the analysis of isotopes of “non-traditional” elements such as strontium allowed a reliable geographic tracing of a variety of food products, since this isotopic signature results from geogenic soil formation that overlies on the geological substrate [33,34]. Few studies have investigated ^87^Sr/^86^Sr ratios in olive oil by means of thermal ionization mass spectrometry (TIMS) [35], as this approach is challenging since the concentration of strontium in olive oil is generally lower than 50 μg kg^−1^ [36]. This critically low concentration is a major obstacle for Sr isotopic analysis. Highly sensitive detection techniques and powerful methods of sample preparation are required to eliminate the matrix and pre-concentrate the strontium.

The main goal of the current review was to summarise the information reported in the literature on (1) the occurrence of inorganic elements and, when possible, light and non-traditional isotopes in olive oils; (2) methods of analysis; and (3) using element analysis to determine geographical provenance. Recent analytical techniques for the authentication of olive oil are reviewed and discussed.

Due to the very low levels of elements reported and the oily and viscous nature of the matrix, its introduction in plasma-based instrumentation is not straightforward. Sample introduction is of paramount importance to prepare for detection. Therefore, we carefully describe and critically discuss sample preparation strategies. Contaminants likely to alter the primary inorganic signatures in olive oil have been identified. Finally, the general multi-elemental and isotopic approaches are also listed. Both the effectiveness and the limitations of these analytical strategies plus detection techniques are discussed, based on the current literature.

## 2. Olive Oil Geographical Authentication by Means of Trace Elements

**Figure 2:**
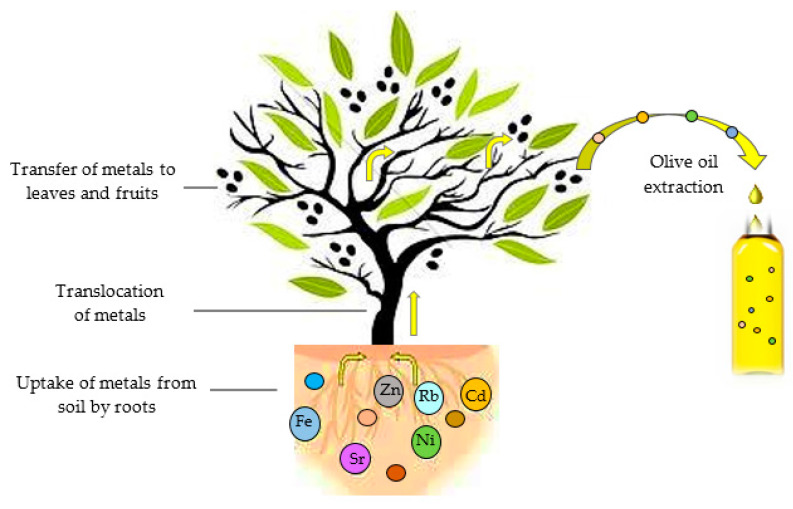
Schematic pathways of how some trace elements arrive in olive oil from the soil.

Trace elements are widely used in the geographical authentication of food products. By definition, elements are trace elements when their concentration in the earth’s crust does not exceed 1 g kg^−1^. They are naturally present in the soil at varying concentrations and are taken up by plants through the roots. In general, it is recognised that the bioavailable elements of the soil are most easily assimilated by plants through the roots and then transferred to the upper parts by translocation [37] (Figure 2). Although several factors can affect element uptake by plants (including the soil pH, the cation exchange capacity, the plant variety, and its age) the elemental composition of a plant mainly reflects soil elemental composition [28,38,39].

Elements are transferred to the soil via the biogeochemical alteration of the rocks. Therefore, the elemental composition of the soil depends mostly on the composition of the bedrock [40]. However, elements can accumulate in soil through anthropogenic activities [39]. Elements can be directly deposited on the soil surface by use of fertilisers and metal-containing pesticides or result from dust fall of industrial activities and automotive traffic (brake dust and exhaust aerosols). These contaminants can be transported by irrigation water and thus can accumulate in plants. The elements assimilated by plants are, therefore, from lithogenic and anthropogenic origins.

Among the elements assimilated by plants, some are essential to growth and development, while others are not. Elements (both essential and nonessential) may also be toxic when present at levels exceeding a certain concentration threshold [28]. For the analysis of elements in olive oil, it is helpful to divide them into three categories: (1) elements such as Sr, Rb and Li are found at varying concentrations in olive oils, although they are not essential for its growth and development. Their concentrations are mainly linked to the type of soil and bedrock. Therefore, they can be used to trace the geological origin [41,42]; (2) Elements such as Cd, Cu and K can be added through agricultural amendments such as the Bordo Mix (mixture of copper sulphate (CuSO_4_) and quicklime (CaO)), and thus they may contribute to link to a region with specific agriculture practices [43]. Cadmium is also a well-known food contaminant that can originate from the use of phosphate fertiliser, or it can be transferred into food through the packaging materials [44] [the composition of the bedrock; (3) Elements such as As, Pb, Cu, Cr and Ni can be sourced from discharges of industrial activities such as textiles and metallurgy.

As shown above, the inorganic signature of food can be an indicator of industrial activities based on a specific geographical area [39]. These findings underline the value of using trace element content to assign geographical origins to food products, and hence of developing a reliable and trustworthy strategy for a precise determination of elemental composition.

### 2.1. Reported Content of Trace Elements in Olive Oil

Until now, compared to other foods, only a few studies have quantified trace elements in olive oil. This is mainly related to the complexity of the olive oil matrix, which makes it tough to develop a reliable analytical procedure.

**Figure 3:**
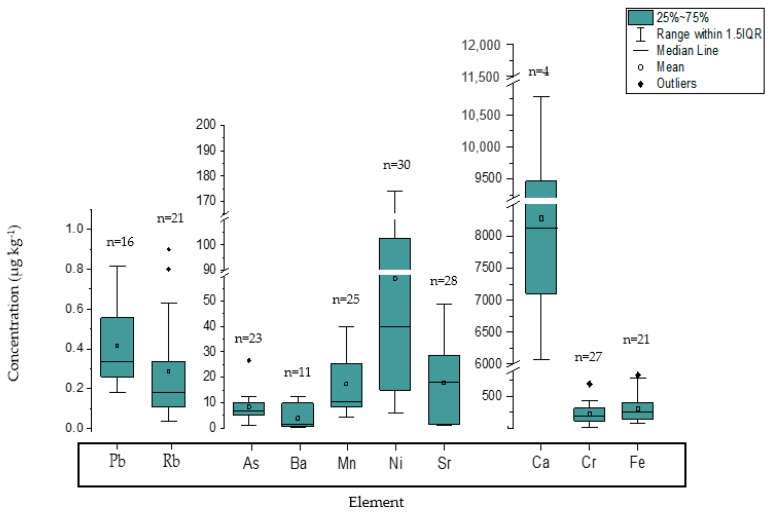
Trace element concentrations in olive oil originating from: Italy, Spain, Tunisia, Portugal, Croatia, Cyprus, Turkey and Greece. (n = number of articles).

The ranges of concentrations of the elements most commonly used for geographical classification are presented in Figure 3. The data presented in this figure are based on the compilation and review of reported results in the scientific literature for olive oils originating from the following countries: Italy, Spain, Tunisia, Portugal, Croatia, Cyprus, Turkey and Greece. Except for Ca, trace elements in olive oils are found at varying concentrations, yet they did not exceed a few hundred micrograms per kilogram (µg kg^−1^). Figure 3 is divided along three vertical axes to form three groups of elements at low, medium and high concentrations. The first category of elements displaying the lower concentration includes Pb and Rb, with median concentrations around 0.25 µg kg^−1^.

The second category includes As, Ba, Mn, Ni and Sr, with medians between >0 and 20 µg kg^−1^, never exceeding 200 µg kg^−1^. Ni shows maximum variation.

The third group of elements, including Ca, Cr and Fe, have been classified as elements at relatively high concentrations, ranging from few hundred µg kg^−1^ up to 10 s of mg kg^−1^; the highest concentrations were reported for Ca.

**Figure 4:**
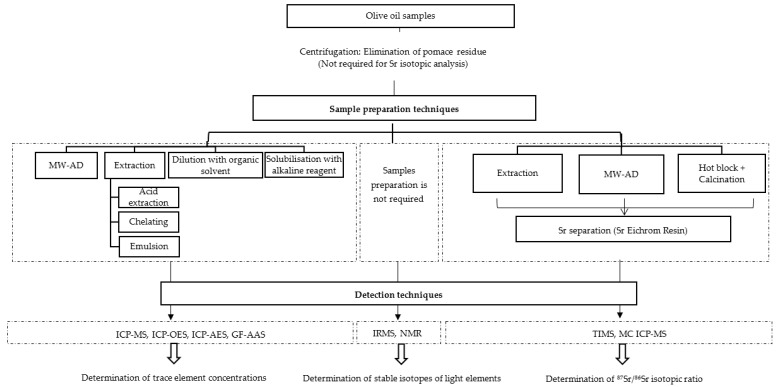
Analytical procedures for the analysis of trace elements, stable isotopes of light elements and Sr isotopic ratio in olive oil.

These elemental concentrations, mostly at critically low values, dissolved in a complex lipid matrix are thus challenging to quantify with high precision. Accordingly, an array of analytical procedures was developed. A wide variety of sample preparation methods were tested, and various detection techniques were employed (Figure 4). The choice of an appropriate analytical strategy is crucial. Nevertheless, critical attention should be paid to the potential contamination that can occur during the sample preparation leading to high blanks and high detection limits. In the case of olive oil, high blanks can be comparable to the elemental concentration of the sample analysed. Accordingly, some elements with initial critically low concentrations, such as Li and Sr [45], or Ni and Cd [46,47], could not be detected or quantified with high precision. This could be one of the main reasons why the concentrations of some elements recently summarised in the literature (Table 1 from Pošćić et al. (2019) [18]) varied enormously. For example, concentrations of Cu and Fe determined in olive oils from Croatia by Zeiner et al. (2005) [48] were three times higher than those reported by Cindric et al. (2007) [49] in oils of the same geographical origins, suggesting that contamination could have occurred during the analytical process.

Similar variability was observed for Li in oils originating from Italy. The reported concentrations ranged from 0.008 to 0.020 µg kg^−1^ [15,41] while olive oils from Spain exhibited a significantly higher concentration of Li, up to 6.4 µg kg^−1^, as reported by Beltran et al. (2015) [45]. This was also the case for Rb in Italian oils. The concentrations recorded were ranging between 0.04 and 0.39 µg kg^−1^ in Italian olive oils [41] and up to 2.6 µg kg^−1^ in Spanish oils [45]. The concentrations of Sr were also found to vary widely between different countries, from less than 0.3 µg kg^−1^ in European oils [15] up to 40 µg kg^−1^ in Tunisian oils [50]. It might be supposed that such variability in concentrations of Li, Rb and Sr can be related to the elemental content of the soil of origin as these elements are mainly originating from it [15,41].

Other element concentrations also varied widely. Co, for examples, was 1.0–5450 µg kg^−1^ [48], Mg was 56–1030 µg kg^−1^ [36], Mn: 3.5–150 µg kg^−1^ [51] and Cr: 12–1830 µg kg^−1^ [52]. Such a large span of elemental concentrations has already raised doubts about the validity of some data in view of the lack of a robust and highly sensitive analytical approach to the detection of elements at critically low concentrations in one of the most complicated food matrices [18]. The published results must be subjected to critical evaluation to avoid comparison with unreliable data, especially in the absence of certified reference materials for trace elements in olive oils. Thus, the validity of the analytical measurements has to be assessed with respect to accuracy and precision. Previously published studies will be discussed below from the general point of view of applied analytical procedures, reliability of obtained results, and their applicability for geographic authentication of olive oil.

### 2.2. Analytical Procedures of Quantification of Trace Elements in Olive Oil

Various sample preparation approaches have been developed for the quantification of trace elements in olive oil. They each aim to release elements from the oily matrix, achieve relatively low limits of quantification (LOQ), and minimise possible contamination. Table 1 summarises the analytical techniques used for the trace elements analysis in olive oil classified according to the methods of sample preparation. Information about the samples analysed, the reagents used for sample treatment, detection techniques, the limit of detection (LOD), the quality control (QC) parameters and the chemometric methods employed are also depicted in Table 1.

#### 2.2.1. Sample Preparation Techniques

Olive oil is a high-viscosity lipid matrix. Its viscosity makes analysis with spectrometric instruments challenging [53]. Due to the critically low concentrations of some elements in olive oil, the latest studies tended to promote the use of ICP-MS known for its high sensitivity.

When using plasma-based ionization techniques, the possible incomplete mineralization of the samples could affect the instrumental performance by forming deposits and clogging parts such as injectors, cones and lenses [54,55]. The presence of traces of unoxidised organic matter could also extinguish plasma sources and thus generates spectral interferences from carbon-based polyatomic ions contributing to the spectral background [56]. Therefore, the development of a reliable sample preparation technique is a crucial step in the process of assessing the elemental composition of olive oil.

Some approaches aim to completely destroy the organic content of the sample and require the use of high-performance digestion equipment under high temperature and high pressure [57]. Others can be implemented under relatively “soft” conditions by involving the use of various organic/inorganic solutions and chemical agents such as chelates, emulsifiers, etc. (Figure 4). They are often designed to obtain a final solution that can be rapidly and easily analysed using various spectrometric instruments [53,58].

(i) Microwave-assisted digestion (MW-AD)

MW-AD is one of the most frequently used sample preparation techniques prior to element analysis for lipid matrices; it is used in approximately 2/3 of the scientific publications in this field (Table 1). A recent review confirmed the reliability and effectiveness of MW-AD for the mineralisation of natural and synthetic oils and oily substances [57]. Organic matter is induced to decompose by high temperature and high pressure [59].

**Table 1:** Determination of trace elements in olive oil and other vegetable oils by means of spectrometric techniques following different sample preparation techniques.

Sample Preparation Techniques	Oil Type/Quality	Number of Samples	Origin	Reagents	Detection Technique	Analytes	Limit of Detection	Material/Method Used for Validation	Accuracy (ACC)/Recovery (R) (%)	Chemometric Method	Purpose	References
**Microwave-assisted digestion**	Extra-Virgin Olive Oil (EVOO)	110	Italy	HNO_3_ and H_2_O_2_	ICP-MS	Ti, V, Cr, Mn, Fe, Co, Ni, Cu, Rb, Sr, Cd, Sb, Ba, W, Tl, Pb, Th, U and REE	-	BCR-668 (musel tissue)	-	PCA, LDA	Authentication, traceability	[60]
	Virgin Olive Oil (VOO)	82	Spain	HNO_3_, H_2_O_2_ and HCl	ICP-MS	Al, As, Ba, Ca, Co, Cr, Cs, Cu, Fe, Ga, Hf, K, Li, Mo, Mg, Mn, Na, Sr, Nb, Ni, Pb, Rb, Sc, Se, Sn, Ta, Th, Ti, U, V, W, Y, Zn and Zr	-	-	-	LDA		[45]
	VOO	36	Italy	HNO_3_	ICP-MS	Be, Mg, Ca, Sc, Cr, Mn, Fe, Co, Ni, As, Se, Sr, Y, Cd, Sb, Sm, Eu and Gd	LOQ: 0.12; 118; 1250; 9.7; 16.3; 9.2; 152; 0.11; 21.2; 0.62; 10.2; 9.6; 0.12; 0.16; 0.14; 0.012; 0.009 and 0.012 (µg kg^−1^)	-	-	LDA		[36]
	Olive oil (OO)	21	Tunisia	HNO_3_ and H_2_O_2_	ICP-MS	Na, Mg, Fe, Zn, V, Mn, As, Rb, Sr, Ba and Pb	0.35; 0.47; 0.12; 0.11 (mg kg^−1^) 1.7; 6; 0.73; 0.3; 5.1; 4.6 and 6.9 (µg kg^−1^)	Multi-element oil standard S23-100Y	ACC: 66–102%	PCA		[50]
	VOO	49	Turkey	HNO_3_ and H_2_O_2_	ICP-MS	Fe, Ca, K, Na, Mg, As, Ba, Co, Cr, Cu, Mn, Ni, Pb, V, Zn	-	-	-	PCA and HCA		[61]
	EVOO	125	Spain	HNO_3_, H_2_O_2_ and HCl	ICP-MS for minor elements and ICP-OES for major elements	Al, Ca, Fe, Mg, Mn, K, Na, Ti, Li, Be, B, V, Cr, Co, Ni, Cu, Zn, Ga, Ge, As, Se, Rb, Sr, Zr, Nb, Mo, Cd, Sn, Sb, Cs, Ba, Hf, Ta, W, Tl, Pb, Bi, Th, U and REE	-	Spike with a multi-element standard solution	R: 82–110%	PCA and LDA		[62]
	EVOO and olive-pomace	1 6 EVOO/10 olive-pomace	Croatia	HNO_3_ and milli-Q water	HR ICP-MS	Li, Rb, Mo, Cd, Sn, Cs, Tl, Pb, Na, Mg, P, S, Ca, Ti, V, Cr, Mn, Fe, Co, Ni, Cu, Zn, Sr, Y, Sb, Ba, La, Ce; and K	-	-	-	-	-	[18]
	VOO, pomace-olive, corn, sunflower and soybean oils	50	Spain	HNO_3_	ICP-MS	Ag, As, Ba, Be, Cd, Co, Cr, Cu, Fe, Hg, Mn, Mo, Ni, Pb, Sb, Ti, Tl and V	0.8; 3.0; 0.5; 1.5; 1.5; 1.5; 8.0; 1.5; 40; 12; 1.5; 1.5; 15; 0.8; 1.5; 15; 1.5 and 2.0 (µg kg^−1^)	109469 Multi-element Standard II Oil Dissolved	R: 85–115%	PCA	Quality identification of oils	[51]
											Discrimination between oils of different types	
	VOO	-	Italy	HNO_3_ and H_2_O_2_	ICP-OES	Pb, Zn, Cd and Cu	-	-	-	-	The influence of olive cultivars and period of harvest on the contents of some elements,	[63]
	OO	90	Tunisia	HNO_3_	ICP-MS	Li, B, Na, Mg, Al, K, Ca, Sc, Cr, Mn, Fe, Co, Ni, Cu, Sr, Mo, Ba and La	0.005; 0.051; 0.104; 5.118; 0.953; 0.319; 0.587; 0.000; 0.000; 0.012; 0.294; 0.005; 0.011; 0.000; 0.006; 0.028; 0.007 and 0.017 (mg kg^−1^)	Spike with standard solutions	R: 69–120%	PCA, LDA and ANOVA	The influence of the irrigation with treated waste water on the multi-elemental profile of olive oils	[30]
**Dry ashing**	OO	17	Croatia	HCl	ElectroThermal Atomic Absorption Spectroscopy (ETAAS)	Cu, Ni, Pb and Fe	-	-	-	-	Comparison between sample preparation procedures	[46]
**Acid extraction**	EVOO	539	Italy	1% HNO_3_/ 0.2% HCl	ICP-MS	Li, Na, Mg, K, Ca, Mn, Co, Cu, Rb, Sr, Cs, Ba, La, Ce, Sm, Eu, Yb, Pb and U	0.005; 40; 14; 60; 30; 0.01; 0.004; 0.13; 0.03; 0.04; 0.003; 0.29; 0.0017; 0.0027; 0.0009; 0.0002; 0.0004; 0.02 and 0.001 (µg kg^−1^)	Spike with NIST 2387 (peanut butter)	R: 82–101%	-	Investigation of mineral composition of authentic PDO Italian olive oils	[41]
	EVOO	267	Italy, France, Spain, Greece and Portugal	6.7% H_2_O_2_ /1% HNO_3_/0,2% HCl	ICP-MS	Li, B, Na, Mg, Al, K, Ca, V, Mn and Co	0.008; 0.17;20; 4; 3; 20; 25; 0.007; 0.2; 0.002 and 0.0006 (µg kg^−1^)	Spike with NIST 2387 (peanut butter) and SPEX s-23 100z	R (NIST): 82–101% R (SPEX standard): 53–92%	Canonical discriminant analysis	Authentication, traceability	[15]
	OO, sunflower, soybean, grape and sesame	-	-	3% HNO_3_	FAAS	Cu, Cd, Ni, Pb and Zn	0.7; 0.3; 0.5; 1.5 and 0.5 (µg kg^−1^)	-	-	-	Development of analytical method	[64]
	EVOO, VOO, ROO, soybean and sunflower oils	-	-	10% HNO_3_	GF-AAS	Cu and Fe	-	Spike with standard	ACC: 94% ± 23–97% ± 12	-		[65]
**Extraction employing an extraction agent**	Sunflower oil, OO, rapeseed oil and salmon oil	-	-	1% Lipase solution at pH 3	ICP-MS	Al, Ba, Cd, Cu, Fe, Mn, Mo, Ni, Ti, V and Zn	0.46, 0.03, 0.007, 0.028, 0.67, 0.038, 0.022, 0.14, 0.17, 0.05 and 0.07 (µg kg^−1^)	EnviroMAT HU-1 Used oil diluted in sunflower oil	R: 83.3–117.8%	-		[66]
	Sunflower oil, OO, rapeseed oil	-	-	0.01 M EDTA solution at pH8	-	Al, Ca, Cd, Cu, Mg, Mn, Ni, Ti, V and Zn,	2.47, 2.81, 0.013, 0.037, 1.37, 0.050, 0.049, 0.47, 0.032 and 0.087 (µg kg^−1^)	Spike of sunflower seed oil with EnviroMAT HU-1	-	-		[67]
	Mustard oil, sun flower oil, sesame oil, ground nut oil, coconut oil, rice bran oil and corn oil	-	-	TMAH and 2% EDTA at pH 12	GF-AAS	Pb, Cd, Cr, Mn, Fe, Cu and Zn	0.6; 0.4; 3.1; 0.3; 0.1; 2.3 and 1.5 (µg kg^−1^)	Spike with analytes	R: 92–97%	-		[68]
**Emulsification**	VOO	5	-	2% Triton X-100	ICP-MS	Al, Ba, Bi, Cd, Co, Cu, Mn, Ni, Pb, Sn and V	5.31; 2.27; 0.98; 0.69; 1.09; 0.33; 0.44; 0.15; 0.02; 0.06 and 3.08 (µg kg^−1^)	Spike with analytes	R: 49.6–176.2%	-		[56]
	Sunflower, hazelnut, canola, corn and OO	50	Turkey	Acidic Triton X-114 solution	ICP-OES	Cd, Cr, Cu, Fe, Mn, Ni, Pb and Zn	-	Spike with analytes	R: 96–109%	ANOVA	Comparison between sample preparation procedures	[52]
**Solubilization with strong alkaline reagent**	Almond, corn, sunflower oils and OO	17	-	TMAH and 1% HNO_3_	ICP-MS	Cu, Ge, Mn, Mo, Ni, Sb, Sr, Ti, V	0.02; 0.05; 0.004; 0.008; 0.012; 0.32; 0.004; 0.28 and 0.02 (µg g^−1^)	-	-	-	Development of analytical method	[69]
**Dilution with organic solvent**	EVOO	50	Spain	Methyl-isobutylketone (MIBK)	Electro-Thermal atomic absorption spectroscopy (ETAAS)	Cu, Cr, Fe, Mn and Ni	25; 1.5; 80; 2 and 10 (pg)	109469 Multi-element Standard II Oil Dissolved	ACC: 97.9–98.75%	Multivariate discriminant analysis	Authentication, traceability	[70]
	Vegetable oils and fats	11	-	Xylene	ICP-OES equipped with hTISIS	Al, Ba, Ca, Cd, Cr, Cu, Fe, Mg, Mn, Mo, Ni, Si, Ti and V	1.6; 0.35; 0.6; 2.6; 0.59; 0.94; 0.86; 0.16; 0.2; 4.1; 2.7; 0.91; 0.21 and 0.81 (µg kg^−1^)	Spike with the Conostan multi-elemental solution	Around 100%	-	Development of analytical method	[53]

The MW-AD method offers many advantages, such as reducing contamination since the solutions are introduced into a closed and sealed system; and reducing the time of mineralization following an optimised program specifically developed according to each type of matrix. Once mineralised, elements are released in an aqueous acidic solution and ready to be analysed after dilution.

However, the MW-AD remains limited for olive oil applications due to the small volume allowed for processing. Generally, the maximum sample amount recommended for olive oil digestion in the most recent MW systems, such as Mars 6 (CEM, https://cem.com, accessed on 16 March 2021), Anton Paar Multiwave (Anton Paar, https://www.anton-paar.com, accessed on 16 March 2021) and Ultrawave (Milestone, https://www.milestonesrl.com, accessed on 16 March 2021), is around 0.5 g.

The resulting mineralised solutions, initially highly concentrated in acid, have to be diluted in order to become suitable for their introduction into an instrumental analysis system. Therefore, after dilution, the concentrations of some elements are likely to be below the LODs. Based on the procedure proposed by Aceto et al. (2019) [60], a high dilution factor of 125 (0.4 g of olive oil transferred into a final volume of 50 mL) would not be adequate for the detection of trace and rare earth elements (REE) in olive oil, given the constraints of LODs imposed by the ICP-MS. Even with a smaller dilution factor (i.e., 50; 0.5 g of olive oil transferred into the final volume of 25 mL), the overall procedure did not allow the determination of Sr in Spanish oils [45]. An alternative procedure was proposed to overcome this issue through the evaporation to dryness of the mineralised sample followed by a re-dissolution in the minimum volume required for analysis [71]. Alternatively, combining samples prepared by evaporation and re-dissolving of a few independently mineralised samples can also be performed [72]. Under these conditions, special attention must be paid to the purity of the reagents used. The solution’s integrity is protected from external contamination by using closed-vessel evaporation systems.

(ii) Ashing in a furnace

The total combustion of olive oil in a furnace (dry ashing) is an uncommon technique and has only been applied to determine selected elements: Cd, Cu, Pb, Fe [73] or Sr, prior to isotopic analysis [35]. The main advantage of this technique is the ability to process a relatively large amount of sample compared to the MW-AD technique, to achieve higher pre-concentration factors. Oil sample amounts ranging from 2 g [46] to 5 g [35] have been treated by ashing in a furnace. Nevertheless, both protocols included a preliminary oxidation step of the oils by the addition of nitric and sulfuric acids and a heating step prior to their introduction into the furnace. All these difficult and tedious procedures may increase analytical and precision errors.

(iii) Methods of extraction
  - Acid extraction (AE)

Acid extraction procedures permit the efficient release of inorganic analytes found in olive oil (i.e., organic phase) into a slightly acidic solution (i.e., aqueous phase). The analytes are released from the oily matrix based on the stronger affinity they have with the acidic solution. Extensive and reliable studies dedicated to the geographic origins of olive oil used this method for years as a sample preparation technique due to its efficiency and relative ease of implementation. The results obtained showed acceptable precision (RSD: 13–27%) and good recovery yields (82–101%) [15,41]. Since then, minor modifications have been proposed, such as using a mixture of diluted acids and the addition of hydrogen peroxide (i.e., 1% HNO_3_, 0.2% HCl and 6.7% H_2_O_2_) [18] to improve and facilitate the decomposition of the organic phase and the release of the elements.

A higher degree of acidification of the aqueous phase (up to 10%) was found to be less efficient for metal extraction [46]. The limitation of the AE method is the relatively low Recovery Rate (RR) compared to the destructive methods [58]. In a recent study published by Poscic et al.; (2019) [18], where AE was applied for the quantification of 29 elements, RR ranged from 60% up to almost 100%, which highlights that elements have different extractability characteristics. Further, it should be noted that the ratios of processed oil to the acidic mixture may vary widely among different studies. Equivalent volumes of oil and acidic mixture were used in the studies performed by Camin et al. (2010, 2010a) [15,41] and Poscic et al. (2019) [18] and led to high RR values, while higher oil proportion (3:1) was found to be less effective, resulting in RR of 20% and 12%, respectively, for Cu and Pb [46]. On the contrary, a micro-extraction of Cu, Cd, Ni, Pb and Zn from 10 mL of olive oil by 200 µL of aqueous solution containing 3% nitric acid showed RR ranging between 95 and 100% [64].

Furthermore, it has been shown that the AE efficiency depends on the duration and temperature of sonication [64,74]. High temperatures tend to reduce the surface tension between two liquids [72] and would alter the emulsion characteristics, i.e., viscosity and droplet size [73]. Under these conditions, the phase mixing would be enhanced and yield a highly effective extraction of the trace elements from oils. Conversely, an increase of the sonication time resulted in a decrease of the extracting efficiency of approximately 15% for Cd [64].

Other parameters have been shown to affect the extraction efficiency. For example, the shape and size of the extraction vessels had a significant effect on the extraction RR since they correlate with the contact surfaces between the two phases [74].

In conclusion, while acidic extraction-based methods have been widely described, the extraction yield cannot be assumed based on previous studies. An optimization of all significant parameters (intensity of shaking, temperature and duration of sonication, volume of extraction reagent…) is mandatory before applying this approach to the extraction of elements from olive oils.

- Extraction using various organic/inorganic agents: chelating and emulsification

The following methods are a slight variation of earlier ones, based on the extraction of analytes with the use of specific extraction agents. The solubilization with a strong alkaline solution [69], emulsification [52,56] and formation of coordination complexes [70,71,72] have been successfully employed for the extraction of trace elements from oils. These approaches offer advantages in terms of sample preparation time and permit the extract to be directly introduced into the ICP. Special attention is often paid to matrix matching with the calibration standards. A summary of the various extraction techniques with their analytical characteristics is presented in Table 1.

(iv) Dilution with organic solvent

Methods involving dilution with organic solvents for olive oil studies [53,70] have the main advantage of being based on the direct analysis of diluted oily matrices (with an adjusted sample introduction system). Recently, a high-temperature torch integrated sample introduction system (hTISIS) was applied for the analysis of oils and fats after dilution with xylene by ICP-OES [53]. The addition of oxygen as an auxiliary gas in ICP-OES was also investigated for that purpose [75]. It is, however, crucial to avoid matrix effects when performing analyses with direct introduction techniques. The time-saving benefit offered by this technique is attractive, but the rigorousness necessary for its implementation and to achieve satisfactory results explain its limited use with respect to olive oil elemental analysis.

(v) Emulsification

Emulsification is an alternative technique to the direct introduction of oil in plasma-based instruments. Emulsifiers are added to oil to form water-soluble micelles. Stabilised “oil-in-water” emulsions can be generated using Triton X-100 and Triton X-114. These emulsifiers were successfully applied for the on-line analysis of olive oil. Triton X-100 was used to extract Tl and Cr, at concentrations above 0.5 µg kg^−1^ and 50 µg kg^−1^, respectively, from about 3 g of olive oil [56]. Triton X-114 was allowed to extract Cd, Cr, Cu, Fe, Mn, Ni, Pb and Zn from olive oil. Their concentrations were determined by ICP-OES, and recoveries ranged from 96% to 109% [52]. During the sample preparation, it was difficult to maintain the stability of the emulsions formed, but stability could be achieved by a thorough control of the experimental conditions [66].

(vi) Chelation of the analytes

The aim of chelation is to form stable complexes (metal/ligand) with higher solubility in the extraction solution. Under appropriate pH conditions, chelation enables the transfer of elements from the oily organic phase to the aqueous extraction phase. Thus, the elements extracted in the aqueous solution would be easily analysed. The chelator can be specific to a single element [76] or an agent that has the ability of complexing a wide range of elements. Examples of chelating extraction using Ethylenediaminetetraacetic acid (EDTA) [66], lipase [67] and a mixture of EDTA/Tetramethylammonium hydroxide (TMAH) [68] have been recently reported. The pH control pH during the extraction phase is of great importance. The extraction yield has been found to be optimal when using lipase at pH = 3, and the mixture of EDTA and TMAH at pH = 12.

This approach is relatively rapid, sensitive and reproducible, and it can be conducted without toxic chemicals, detergents and concentrated acids. However, the reagents must have a high degree of purity.

All the above-mentioned methods have shown promising results and are good choices for future experiments. Some of them are relatively rapid as no pre-treatments are required (i.e., emulsification, chelating and direct dilution), while others are simple to perform (MW-AD and acid extraction). Some of the methods require specific equipment (i.e., direct dilution). The choice of method will depend on the research targets and the availability of the apparatus and reagents. Since, in general, the concentrations of trace elements in olive oil are low, methods that allow a pre-concentration with controlled purity reagents are the most suitable.

(vii) Comparative studies of different sample preparation methods

As discussed before, each sample preparation technique has its advantages and limitations. The first reliable comparison between MW-AD, AE and dry ashing for olive oil samples has demonstrated that only the latter allows the determination by ET-AAS of all elements of interest [46]. Two recent studies have also assessed the robustness and efficiency of the most common MW-AD and acid extraction procedures [18,71]. Results published by Damak et al. (2019) showed that the ultrasound-assisted AE achieved lower LODs, higher precision and better repeatability than MW-AD combined with evaporation [71]. The advantage of the AE approach over the MW-AD was also highlighted by Pošcic et al. (2019), who stated that the MW-AD is limited for the measurement of very low concentrations, as the case of trace elements in olive oil [18]. Nevertheless, in both studies, it was reported that there was no significant difference between concentrations found after using both techniques for the quantification of trace elements in olive oil. Therefore, on the basis of these findings, it would be advantageous to perform AE in order to quantify more easily trace elements in olive oil and other matrices with similar characteristics.

The review of the current scientific literature for the quantification of trace elements in olive oil shows that the choice of an appropriate sample preparation method is crucial as it can affect the detection of analytes and the quality of the results. The choice of method should be based on the objectives of the research study and on the analytical performance of the instrumentation to be used for the detection stage.

#### 2.2.2. Detection Techniques

**Figure 5:**
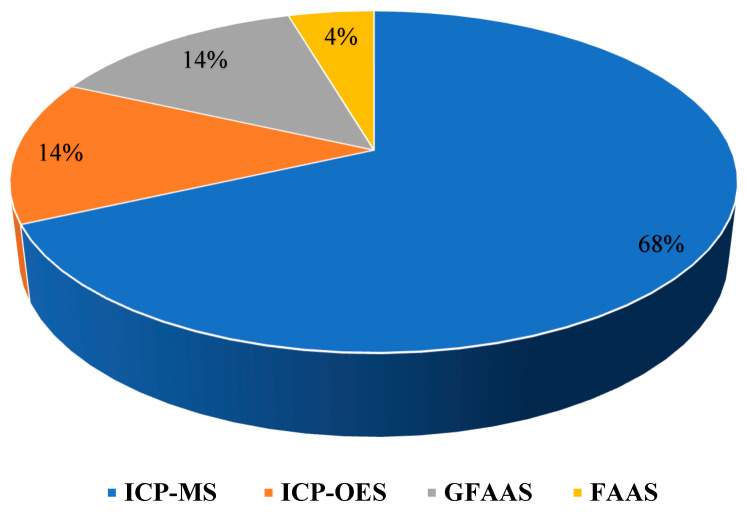
Percentage of articles that use atomic spectroscopic techniques to analyse trace elements in olive oil.

The quantification of trace elements in olive oil requires sensitive detection techniques. The ICP-MS is known to be one of the most sensitive instruments available today for the accurate quantification of trace elements. Accordingly, and based on our literature survey, the main analytical instrument used to determine the elemental content of olive oil for geographical traceability was the ICP-MS (almost 70% of the published studies, Figure 5).

Other spectroscopic methods, such as ICP-OES, GF-AAS and FAAS were mostly used for the detection of alkaline metals in olive oils as these are present at higher concentrations [58]. The plasma-based detection techniques (OES or MS) now occupy the leading position mainly when extraction [68], emulsification [52], liquid-liquid extraction [64] or dilution with organic solvent [70] are used as sample preparation techniques. However, one-third of studies reviewed (Table 1) did not provide information on detection limits of analysed elements limiting the reader’s capacity to assess the validity and credibility of the results. Some authors have reported surprisingly high LODs and LOQs for ICP-MS, such as 6 µg kg^−1^ (Mn) and 6.9 µg kg^−1^ (Pb) [50]; or 9.6 µg kg^−1^ (Sr), 16.3 µg kg^−1^ (Cr) and 21.2 µg kg^−1^ (Ni) µg kg^−1^ [36].

In the absence of information about the measured procedural blank, it is difficult to explain such atypical high LODs. It can only be hypothesised that these LODs are most likely due to possible contaminations during sample preparation procedures.

#### 2.2.3. Analytical Method Validation

Both precision and accuracy are important parameters of the method validation procedure. For geographical traceability studies based on the elemental content, a difference between the measured and the expected concentration of analyte that would be typically described as non-significant or classified as an analytical error would distort the results and lead to false assumptions and wrong geographical classification of the studied samples. Especially for olive oil authentication, as trace elements are initially at low concentrations, the difference in the elemental profile between distinct geographical origins is likely very limited. Thus, it is crucial to determine the element concentrations with a high degree of precision and accuracy to be able to perform a proper assessment of the origin.

Thus far, there is still no certified reference material (CRM) for trace elements in olive oil despite of its growing worldwide consumption. This is the reason why a number of authors did not use CRMs in their experiments, or at least why such information was not provided [36,45,61,63,64,69].

However, those who have attempted to evaluate the performance of their analytical method have often aimed to use a CRM that would have physicochemical properties close to those of olive oil. The most commonly used ones are based on engine or synthetic oils, such as the multi-element organometallic oil standard on base oil (S23-100Y from SPEX CertiPrep) [15,50] which is an oil for mechanical engines. There is also a Multi-element Standard II Oil Dissolved from Certipur^®^ (109469) [51,70], and a used oil CRM (EnviroMAT HU-1) from SCP Science [66,67]. It must be mentioned that these CRMs do not have the same viscosity as olive oils and the concentrations of certified elements are not comparable to those of olive oil. The closest CRM of natural edible lipid matrix available is the certified peanut butter (NIST SRM 2387). It has been seldomly used either diluted in oil or in its raw state by some researchers [15,41,47]. This CRM is characterised by its relatively high elemental concentrations compared to olive oil, which makes it more suitable as a spiking standard for olive oil. Recently, Aceto et al. (2019) used an uncommon CRM, BCR-668 muscle tissue, in order to quantify lanthanides and other microelements distribution to authenticate Italian extra-virgin olive oils after microwave-assisted digestion [60]. However, the choice of such biological tissue is quite controversial, especially in the case of olive oil analyses, as the levels of REE in the CRM are at least three times higher than those in olive oils. Further, the main constituents of olive oils and muscle tissue matrices are different qualitatively and quantitatively. Therefore, serious discrepancies related to matrix effects could be expected. Unfortunately, the authors did not provide justifications for their choice of CRM. Indeed, the inconsistency between the calibration standard concentration range selected and the concentrations of the expected elements in olive oil samples also suggests that the results could be biased.

Furthermore, an array of studies has determined the elemental recoveries by spiking each individual oil sample with a multi-elemental standard solution. This approach is acceptable when the selected sample preparation method involves the complete destruction of the organic matter such as MW-AD [30,62] or ashing in the furnace [46]. However, despite using mono- or multi-elemental spiking solutions for validation purposes for the extraction [68,74] or the emulsification methods [52,56], it is very unlikely that this approach reflects the real extraction efficiency of an element initially present in the oily phase, since the elements composing the spiked solutions are already in a water-soluble form and no extraction is required. It is, therefore, practically impossible to define a realistic target recovery using a spike of elements in an aqueous solution, and it could even be misleading to do so using this approach.

In conclusion, despite the unavailability of a suitable CRM for olive oil, a wide range of alternative methods have been proposed to ensure quality assessment. The use of synthetic oils and similar lipid matrices certified for trace elements or spiking with natural oils seemed appropriate. They allowed effective control of the measurement performance pending the development of an appropriate matrix-matched CRM for olive oil.

### 2.3. Limits of Olive Oil Authentication by Means of Trace Elements

Trace element contents in plants are mainly related to the soil composition where they were grown. However, correlations between the elemental content in oil and the corresponding soil are not always valid. Many factors may influence the elemental composition of olive oil through changes in the fruit composition during its growing and ripening period or through external contamination during its extraction and packaging.

#### 2.3.1. Influence of Agro-Climate Conditions

It has been demonstrated that several parameters such as the olive cultivar [63], irrigation of olive trees [30], use of fertilisers [77], or fluctuations in annual climatic parameters [78] may modify the elemental profile of olive oil. At present, no consensus has been reached with regard to the possible existence of a direct and clear correlation between the concentration of trace elements in the soil and in the paired olive oils. However, interesting findings have recently emerged. Damak et al. (2019) sampled olives and soils from various Tunisian regions in order to investigate the correlation between the elemental content of soil and that of olives [50]. After olive oil extraction from olives, its elemental content was determined by ICP-MS after MW-AD, while the soil content was analysed by X-ray fluorescence (XRF). The XRF beads were also analysed for trace elements by femtosecond ultraviolet Laser Ablation ICP-MS. The results of this study have demonstrated that the correlation between the composition of the soil and that of the plant is not always straightforward. The concentration of the elements analysed (Fe, Mn, Zn, Na and Pb) in olive oil were inversely proportional to those recorded in the soils. Based on these results, the authors concluded that the soil is not the only source of trace elements in olive oil. On the basis of these results, it can also be suggested that the results were influenced by the different sample treatments of oil and soils and the different analytical performances of the detection instruments used.

According to Greenough et al. (2010) [78], the multi-elemental composition of an agri-food product is influenced by the climatic conditions (temperature, precipitation, humidity) prevailing during its growth. A recent study of Portuguese olive oils by Gouvinhas et al. (2016) [79] showed that trace element content could vary according to the ripeness of the olives. Another study suggested a correlation between Fe, Co, Ni, Ba, Rb, Sr and Pb in the soils and olive oils [60], but elements such as W, Tl, Cr, Sb, Cd and Cu exhibited a different trend; the best correlation was observed for the lanthanides. Since the lanthanides are adsorbed passively from soil and are not altered much when passing from soil to fruit, they may act as a promising tracer when analytical methods enable detection at the ultra-trace level.

#### 2.3.2. Influence of the Olive Oil Extraction Process

After harvesting, the collected fruits will undergo several steps that may alter the mineral composition of the final product (Figure 1). During the oil extraction process, olives are in direct contact with metallic surfaces that can generate contamination in the final product.

Further, the possible addition of water after mixing and prior to centrifugation to increase the oil extraction efficiency may be a potential source of contamination with trace elements. These concerns have been evaluated by Sandeep Banerjee (2015), who showed that the use of water during the oil extraction process could modify its elemental composition [80]. He reported that the concentration of Sc, Cr, Fe, Co and Ni in the oil increased with the addition of water. On the other hand, the use of water at the industrial-scale olive oil refining process decreased the Ca concentrations in olive oils.

Recently, it has been demonstrated that the filtration process of olive oil, frequently required to remove the pomace residues, may significantly alter the trace element content of both olive oil and pomace residues [81]. In addition to the changes that will affect the sensorial and physicochemical characteristics of the oil [82], Pošćić et al. (2019) recently demonstrated that the pomace residues significantly affected the elemental content of the oil [18]. In their study, 29 element concentrations were determined in centrifuged and non-centrifuged olive oils. The results were expressed as NC-to-C (non-centrifuged vs. centrifuged) ratios for each element analysed, and displayed discrepancies of up to three orders of magnitude. This fact needs to be specifically considered for accurate quantification of trace elements in olive oil. According to Pošćić et al. (2019), a centrifugation step is mandatory prior to trace element analysis. Without centrifugation, the results are not reliable enough for comparison purposes [18]. Finally, the storage and transportation conditions of olive oil would affect the final chemical composition. De Leonardis et al. (2000) suggested the use of polypropylene containers when determining levels of Cu and Fe in edible vegetable oils [65].

All the above-mentioned factors can alter the inorganic signature of olive oil and should be carefully investigated to assess the geographical origin of the oils.

## 3. Olive Oil Geographical Traceability by Means of Isotopic Analysis

Since the inorganic oil composition does not always match that of the soil when using multi-elemental signatures, stable isotopes have long been used for traceability purposes in foods [83]. The earliest uses of the stable isotope analysis technique to establish the authenticity of foods dates back to the early 1970s [84]. The isotopic ratios of some light elements could change depending on the geographical origin, the climatic parameters, and finally, the soil paedology and geology of the location from where the products originate [85].

### 3.1. Stable Isotopes of Light Elements (C, H and O)

The use of stable isotope ratios of hydrogen, carbon and oxygen provides information on the geographical origin and the pedo-climatic context. The major elemental constituents of the olive oil (H, C, O) have different stable isotopes (i.e., ^1^H, ^2^H; ^12^C, ^13^C; ^16^O, ^18^O). The relative abundance of each of these stable isotopes could vary in the environment, depending on (i) the origin of the elements and (ii) the processes that will modify their concentrations.

A number of scientific studies have aimed to use the combination of the isotopic ratios of light elements for the geographical determination of the production origin of olive oils and their potential adulteration [41,86,87,88,89,90].

Carbon and oxygen isotope data are expressed in the usual delta notation (δ^13^C and δ^18^O), defined as:

δ_sample_ (‰) = ((R_sample_/R_standard_) − 1) × 1000 (1)

where R is the isotopic ratio ^2^H/^1^H, ^13^C/^12^C or ^18^O/^16^O. The international standard for C is Pee Dee Belemnite (PDB) while the Standard Mean Ocean Water (SMOW) is used for O and H.

The raw isotopic analyses of the samples are corrected by means of internationally-accepted matrix-matching reference materials. The most appropriate certified reference for olive oil are NBS22, USGS84 and USG85 [80,91].

In general, for the studies focused on olive oils, the main stable isotopic ratios used were ^13^C/^12^C, ^18^O/^16^O and ^2^H/^1^H.

(i) δ^13^C measurements as a tracer of physiological processes of the plant

δ^13^C measurements have been used for decades as a tracer of the type of photosynthesis (C3, C4, CAM) [92]. The fixation and assimilation of atmospheric CO_2_ during photosynthesis varies according to the metabolic pathway through the mechanism of glucose production. The ranges of δ^13^C variations for C3 plants vary from −32 to −24%, from −16 to –10% for C4 plants, and from to −30 to −12% for CAM [93]. These significant differences have been used at first to reconstruct past and paleo-environments [94]. Further, they can also be used to detect the adulteration of food products. A common fraudulent practice in food is sweetening products with cheap sugar by the addition of C4 sugar to C3 crops; this can be easily detected by determining δ^13^C values [95]. More recently, it has been shown that genetic determinism is likely to influence the variation the δ^13^C values as well, resulting in oscillating values close to the typical photosynthetic signature [32,89,96].

The δ^13^C values can also be affected by the developmental stage of the plant [89]. In the specific case of olive trees, it has been applied to evaluate the olive ripeness index. These developments using the isotopic signatures of light elements of olive oils were further highlighted by S. Portarena [89]. The results obtained in this study demonstrated a positive correlation between the C and the O isotopic compositions and the plant maturity stage. This was also valid to establish the evolution of the climatic changes during the fruit-ripening season and more especially during the period of maximum oil accumulation.

(ii) δ^2^H and δ^18^O measurements as a tracer of water dynamics

The hydrogen and oxygen isotope composition of water (δ^2^H and δ^18^O) will vary depending on (i) the geographical position; the latitude, the longitude, the elevation, the distance from the sea, (ii) the type of climate; amount and type of precipitation, evaporation, (iii) type of plants which impact combining with the climate the rate of evaporation by plant transpiration.

Several studies have demonstrated that stable isotopes of light elements are good indicators of geographic origin. Table 2 summarises the different studies that have successfully used stable isotopes of light elements for olive oil geographical authentication. Analyses are performed using an isotope ratio mass spectrometer (IRMS) generally connected to a high-temperature source for combustion or pyrolysis of olive oil, typically without prior pre-treatment.

A rapid and cost-effective pre-treatment methodology for measuring C and O isotopic ratios in oil and glycerol was proposed. The glycerol was obtained by distillation after hydrolysis and extraction of the fatty acids. A correlation was found between C in oil and in glycerol extracted (δ^13^C glycerol = 1.1114 × δ^13^C bulk oil + 1.4057; *p* < 0.001) [41]. Moreover, δ^18^O of glycerol is correlated with the δ^18^O of leaf water. These promising results indicate that the direct measurement of isotopic ratios in glycerol would be beneficial.

The measurement of the natural stable isotopic composition of light elements allowed the tracing of several environmental conditions. However, since the plants adapt to their specific environment, the isotopic variations may be very small for the same type of plants (photosynthesis) when the variations of environmental conditions are not significant between different study areas. Camin et al. (2016) [31] assumed that these similarities between the different locations could result from similar climatic conditions in both regions during the pre-harvesting period. Therefore, geographically distinct regions could nonetheless display identical isotopic ratios but only if the climate is equivalent in the two different locations. To further reinforce the geographical discrimination, the combined use with ^1^H NMR data using a multivariate statistical approach was investigated.

Recently, Tarapoulouzi et al. (2021) conducted a large-scale study on a total of 100 monovarietal olive oil samples from two coastal locations in Greece [90]. The sampling strategy aimed to avoid the effect of olive oil storage and seasonal changes. The combination of stable isotopes of light element (δ^13^C, δ^18^O and δ^2^H) with a chemometric tool (OPLS-DA) enabled the separation between the samples from (i) two geographical areas, (ii) three cultivars, two of which belonged to the same geographical area. Based on these results, the olive cultivar was more effective for the samples classification compared to the geographical origin.

Further, the chemometric analysis showed that the discriminating potential of stable isotopes of H and O was significantly higher compared to C isotopes since the sampling areas shared similar climatological and geographic characteristics. Accordingly, the choice of appropriate isotopic approaches for geographical traceability studies should be based on the plant type and genetic determinism, the geographic, environmental and climatological characteristics of the plant growing area.

A large international database of the range of stable isotope ratios of light elements was established based on the published results obtained in various geographical locations. Nevertheless, Camin et al. (2010) highlighted the significant variation in the ratios ^13^C/^12^C and ^18^O/^16^O of Italian olive oils sampled from 2000 to 2005. The isotope ratios of the samples collected from the same region varied among the years, characterised by different climatic conditions (rainfall and temperature) [41].

**Table 2:** Determination of isotopic ratios of light elements in olive oil and other edible oils.

Samples	Number of Samples	Origin	Isotopes	Detection Technique (Manufacturer)	Statistical Evaluation	Complementary Analysis	Purpose of Using Isotopes	References
EVOO	539	Italy	^18^O/^16^O, ^2^H/^1^H and ^13^C/^12^C	IRMS (Finnigan DELTA XP, Thermo Scientific, Bremen, Germany)	The non-parametric test of Kruskall–Wallis	Multi-elemental analysis	Discrimination capability of ^18^O/^16^O, ^2^H/^1^H and ^13^C/^12^C between olive oils from different Italian regions. The trend of these indicators over the years.	[41]
EVOO	267	Italy, France, Spain, Greece and Portugal	^18^O/^16^O, ^2^H/^1^H and ^13^C/^12^C	IRMS (Delta plus XL, Delta Plus XP, Delta V, Delta S, Thermo- Finnigan, Bremen, Gremany; Isoprime, AP2003, GV Instruments Ltd., Manchester, U.K.; Optima Micromass)	Kolmogorov-Smirnov test		Finding correlation between H, C and O isotope ratios in olive oil and climatic and geographical characteristics of the provenance locations.	[15]
VOO	49	Turkey	^13^C/^12^C	IRMS (Micro- mass, IsoPrime)	PCA and HCA		Combination of trace element concentrations and ^13^C/^12^C isotope ratio for better resolution in geographical discrimination of olive oils.	[61]
VOO	47	Greece	^18^O/^16^O and ^13^C/^12^C	IRMS (Finnigan Delta V Advantage, Thermo Fisher Scientific, Bremen, Germany)	Multivariate analysis	Multi-elemental analysis/ Determination of chlorophyll and carotenoid pigments	Combination of ^18^O/^16^O and ^13^C/^12^C isotope ratios and physicochemical parameters for geographical classification of olive oils from regions in proximity.	[97]
VOO	387	Italy	^18^O/^16^O and ^13^C/^12^C	IRMS (Isoprime, Cheadle, UK)	Pearson coefficient and ANOVA	-	Use of stable isotope ratios as tracers for environmental conditions and geographic coordinates for olive oil geographical authentication.	[32]
Edible oils and sweeteners	43	Italy, Greece and Spain	^18^O/^16^O, ^2^H/^1^H and ^13^C/^12^C	IRMS (Thermo-Finnigan Delta plus XP, Thermo Fisher Scientific Inc., Waltham, MA, USA)	-	Multi-elemental analysis	Use of carbon isotope ratio as indication of olive oil adulteration (with corn oil). Oxygen and hydrogen isotope fractionation between edible oils (olive oil) and local meteoric water.	[80]
OO	180	Italy	^18^O/^16^O, ^2^H/^1^H and ^13^C/^12^C	IRMS (Finnigan DELTA XP, Thermo Scientific, Bremen, Germany)	-	The acidity values, UV spectrophotometric indices (K232, K270, DK) and fatty acid composition	Measurement of Stable isotope ratios in legal applications for geographical origin of food (olive oil).	[31]
OO	-	Italy	^18^O/^16^O and ^13^C/^12^C	IRMS (Isoprime, GV, Cheadle, UK)	Factorial analysis of-variance (ANOVA) and Post Hoc Fisher multiple comparison test	Fatty acid composition	Effect of the cultivar and the ripening stage of olives on C and O isotope composition for traceability studies.	[89]
EVOO	53	Italy and Croatia	^18^O/^16^O and ^13^C/^12^C	IRMS (Delta plus XP; ThermoFinnigan, Bremen, German)	Linear discriminant analysis	Major chemical component determination (triacylglycerol and fatty acids)/Thermal properties	Comparison between conventional techniques, stable isotope ratio analysis and thermal properties for olive oil traceability resolution.	[98]
OO	100	Greece	^18^O/^16^O, ^2^H/^1^H and ^13^C/^12^C	IRMS (Nu Instruments Limited, Wrexham, UK)	PCA/OPLS-DA	-	Creation of OPLS-DA model, using stable isotope ratios of C, H and O in olive oil, able to discriminate and predict origin of samples from different origin.	[90]
EVOO	210	Greece	^18^O/^16^O and ^13^C/^12^C	IRMS (not mentioned)	-	-	C and O isotope ratios as markers of the climate regime thus of the geographical origin of olive oils. Use of ^13^C isotopic values of biophenolic extracts for geographical discrimination of olive oil.	[88]

Therefore, the variable nature of the environmental and climatic conditions over the years requires a continuous update of isotopic data that evolve consistently.

### 3.2. Strontium Isotope Ratio Determination for Olive Oil Geographical Traceability

Strontium isotopic signatures are widely used for food traceability and many other applications where issues of origin are important. Strontium is relatively abundant in soil, is not metabolised by living organisms and does not seem to undergo any detectable fractionation. This results in a fully conservative behaviour of the Sr isotopic ratio from the soils to the plants, including animals feeding these plants [99]. Strontium is an alkaline earth metal naturally present in rocks, soil and water, and has four stable isotopes (^84,86,87,88^Sr). The ^87^Sr isotope is radiogenic as it is derived from the disintegration of ^87^Rb. The isotopic ratio ^87^Sr/^86^Sr most often considered in studies is related to the intrinsic nature of the bedrock, its age, and its geological evolution and is, therefore, specific for each type of soil. The two main sources of this element in plants are the labile Sr fraction of the soil, and the Sr contained in soil pore water [100]. Strontium is absorbed by the plant and distributed into its components from the soil without undergoing isotopic fractionation [34].

Given these features, strontium has been successfully used for the geographical tracing of different food products such as wheat [101], wine [102], coffee [103] and fruits such as orange [104]. Recently, strontium isotopic signature was used to assess the geographical traceability of olive oil. Benincasa et al. (2007) were the first to report the use of strontium isotopic ratio measurement in olive oil for traceability purposes, but the complex nature of the matrix and the low Sr content (<50 µg kg^−1^) are challenging for most analytical methods [36]. Consequently, pre-concentration is mandatory to achieve suitable results. 

S. Medini et al. (2015) [35] developed an analytical method for the measurement of ^87^Sr/^86^Sr in olive oil for authenticating AOP olive oil from southern France. As a pre-screening step, the elemental Sr content in the samples was determined using ICP-MS after MW-AD. The strontium concentrations ranged between 2 and 13.9 µg kg^−1^, which are critically low for accurate measurement of the isotopic ratio of Sr by mass spectrometry. Therefore, ashing of the oils in a muffle furnace was performed, which allowed to increase the amount of sample that can be processed. The Sr purification was performed using Sr specific resin (Sr Eichrom resin) according to the protocol described by Pin et al. (2003) [105]. Finally, the Sr isotopic ratios were determined by thermal ionization mass spectrometry (TIMS) in five olive oils from Nîmes PDO (France) and in two olive oils from Morocco. The results showed that olive oils from France and Morocco were successfully distinguished according to Sr isotopic ratios and were correlated to the bedrock geology. Good reproducibility of the extraction method used was also demonstrated. Given the unavailability of a reference material certified for the isotope ratio ^87^Sr/^86^Sr in olive oil, a strontium carbonate isotopic CRM (NIST 987) was subjected to the same procedure that olive oil samples to study the influence of pre-treatments and calcination on ^87^Sr/^86^Sr ratio. The results demonstrated that the proposed methodology did not alter the isotopic signature of Sr. However, the influence of the procedure on an oily matrix has not been demonstrated since NIST 987 is an aqueous solution with a high degree of homogeneity that does not reflect the complex nature of the olive oil matrix.

Techer et al. (2017) [106] studied the impact of agricultural practices on the Sr isotopic composition of olives. TIMS was used to determine the Sr isotopic compositions (^87^Sr/^86^Sr) of varied parts of olives trees (branches, leaves and olives) and the Sr isotopic composition of their growing environment in two distinct agricultural contexts belonging to the same geological area. The aim of the study was to assess the impact of the irrigation and fertilisation techniques on the Sr isotopic composition of olive trees. The study showed that using anthropogenic additives and irrigation water induced changes in the composition of Sr in the soil and thus in the plant. This study underscored the importance of Sr as a reliable tool for the geographical traceability of olive oil.

The different studies based on the use of isotopes of light elements for olive oil geographical discrimination showed that analyzing a combination of δ^13^C and δ^18^O of organic plant matter can be a powerful tool. This approach integrates the ecophysiological behaviour, which itself is directly related to the type of photosynthesis and the pedoclimatic context. Sr isotopic signatures have been very useful to trace the geographical origin of different agricultural products, since they are usually conservatively up taken by the plants from the soils they grow in and are stored in the plant with little to no detectable isotopic fractionation. The use of stable isotope ratios of light elements (C, H and O) permits tracing the pedoclimatic conditions in the growing area of the plant, while the use of strontium isotopes provides information on the geological substratum. Therefore, it would be relevant to combine the two isotopic approaches to provide accurate and complementary information on the geographical location of the production area.

## 4. Statistical Analysis for Geographical Authentication of Olive Oil

To summarise and better exploit the data in a study of geographical authentication of olive oil, a statistical representation is generally used. The most common statistical methods that have demonstrated discrimination between olive oils originating from different geographical origins are multivariate analyses, mainly Principal Component Analysis (PCA) and Linear Discriminant Analysis (LDA) [107]. PCA is an unsupervised method, and LDA is supervised. The PCA score plot is a graphical representation of variables called principal components [108]. PCA maintains the variability of data. In most cases, a PC1 vs PC2 score plot applied to trace element content worked well to distinguish between olive oil samples of different origins [60,61,62]. Nevertheless, PCA applied to mineral composition is not always sufficient. For example, olive oils from 12 different geographic locations in Spain could not be distinguished only using trace elements [62].

With PCA, graphic representation of two principal components separated the samples into two groups, either belonging to a cluster of points representing a common geographical origin (Huelva) or to one of the other regions, which were then separated into three main clusters distinguished by LDA. A similar conclusion has been drawn for the geographical authentication of virgin olive oils from Western Turkey [61]: the PCA method was not able to distinguish Turkish olive oils from different locations. Only two groups of two sets of samples were successfully classified by means of a score plot, each group containing samples from different locations. This study was enhanced by the use of δ^13^C, which can further distinguish sample groups by providing additional information to resolve similarities between some element content in different samples. It was demonstrated that stable isotopes of light elements can be successfully coupled with the mineral composition of the oil to better pinpoint the geographic origins of different olive oils.

As for PCA, LDA helps to summarise the main results on a two-dimensional plot while trying to maximise the discrimination between known groups. The sample classification is based on a maximum likelihood discriminant rule. To study the geographical traceability of olive oil from southwestern Spain, trace element concentrations in soils, olive pomace, and olive oils were determined [45]. LDA was successfully applied to the concentrations of Fe, Na and W to discriminate soils from four different geographical locations. Then, since trace element concentrations were lower in the olive pomace, only eight element concentrations (i.e., W, Mg, Mn, Ca, Fe, Ba, Li and Na) were used by the LDA for optimal classification. Despite the overlap of some points, it is still possible to discriminate between four groups of points representing the different geographic locations. This classification was less effective for olive oil using the eight aforementioned elements due to their very low concentrations in the oil. Indeed, the critically low concentrations in such a complex matrix may not be measured with high precision (problem of LOD, sensitivity, repeatability), which makes this statistical method limited for the geographical discrimination of olive oil. In this study, Mn concentration in olive oils ranged from 0.1 to 0.2 µg kg^−1^, while the lower concentration of calibration standard solutions used was equal to 0.2 µg kg^−1^. That makes the measured concentration unreliable, especially as Mn was used by LDA. Thus, if the analysis of trace elements in the soil allows for good geographical discrimination, this does not necessarily reflect the same distribution for the corresponding olive oils.

On the other hand, in a study published in 2007, LDA applied to Italian olive oils showed a remarkable discriminating power on the basis of 18 elements. A total of 8 groups of samples were clearly differentiated on an LDA plot with high reliability [36]. PCA can explain the distribution of groups of samples according to the influencing elements. It first identifies the elements, then identifies the possible interactions and combinations between them. LDA is the best alternative in the case of PCA failure to find a high enough variance among the principal components (as, for PCA, this induces a loss of information when reducing the dimensions).

## 5. Conclusions

This review summarises the developments in inorganic and isotopic analysis for geographical authentication of olive oil. The first and foremost observation based on the compilation of inorganic content in olive oils is that, in general, except for elements such as Fe, Cr and Ca, elements are at trace and ultra-trace levels well below 200 µg kg^−1^ on average. The analytical isotopic characterization of this matrix remains challenging.

Recent developments in sample preparation techniques and spectroscopic/spectrometric analysis have improved the quality of food science. However, the adaptability of these methods to the olive oil matrix remains limited. Various analytical methods have been proposed in the literature to ensure the geographical traceability of olive oil, an economically important product; most of these methods operate via destructive sample preparation strategies (mainly microwave-assisted digestion), aiming to eliminate the organic matrix, which is a barrier to the introduction of samples in spectroscopic analysis instrumentation. However, the destruction of organic matter is often incomplete, leading to unreliable results. In addition, the absence of certified reference material for olive oil remains a significant concern, limiting the reliability of the published results and hindering the validation of the proposed methods.

Non-destructive methods have also been developed to release the elements to be analysed without totally destroying the organic matrix. These are typically extraction/complexation methods that are generally more labour-intensive, although they are performed under gentle conditions. The reproducibility of results and the extraction yield remain an analytical challenge. In general, ICP-MS is the most appropriate detection technique for the quantification of elements at low concentrations. Very strict control of the blank throughout sample preparation procedures is, however, required. If not fully controlled, LODs can be high.

Further to the sample preparation and detection procedures, multivariate statistical models, both supervised and unsupervised, are generally applied to multi-elemental profiles of oils. They have shown that it is possible to classify olive oils according to their geographical origin on the basis of reliable data. Nevertheless, multi-elemental analysis remains limited for the identification of the geographical origin of olive oil, as various external factors can alter the element concentration.

In addition to the multi-elemental profile, isotopic approaches can complete the desired information on geographic origin. Stable isotopes of light elements have provided additional information on the plant’s growing environment, but these alone are not enough to confirm proper geographical authentication. They give information about the environmental and climatic characteristics of locations and the physiology of the plant. All these aspects are likely to modify the isotopic profile of light elements. More recent developments in the analysis of the Sr isotope ratio in olive oil have enhanced the traceability studies of oils. The isotopic ratio ^87^Sr/^86^Sr is directly and exclusively related to the geology of the soil and does not fractionate from the soil to the final product, thus, it maintains the isotopic signature of the original soil. However, the critically low concentration of Sr in olive oil makes it a serious analytical challenge for its extraction from the matrix, its precise quantification, and the determination of its isotopic ratio. Further studies on the pre-concentration of Sr in olive oil are required to strengthen this approach.

## Data Availability

All data mentioned were compiled from research articles that have been listed in the literature section.

## Sample Availability

Samples of the compounds are available from the authors.
